# Ceftriaxone Pulse Dosing Fails to Eradicate Biofilm-Like Microcolony *B. burgdorferi* Persisters Which Are Sterilized by Daptomycin/ Doxycycline/Cefuroxime without Pulse Dosing

**DOI:** 10.3389/fmicb.2016.01744

**Published:** 2016-11-04

**Authors:** Jie Feng, Shuo Zhang, Wanliang Shi, Ying Zhang

**Affiliations:** Department of Molecular Microbiology and Immunology, Bloomberg School of Public Health, Johns Hopkins UniversityBaltimore, MD, USA

**Keywords:** *Borrelia burgdorferi*, persisters, biofilm, pulse dosing, drug combination

## Abstract

Although the majority of Lyme disease patients can be cured, at least 10–20% of the patients continue to suffer from persisting symptoms such as fatigue, muscular and joint pain, and neurologic impairment after standard 2–4 week antibiotic treatment. While the causes for this post-treatment Lyme disease symptoms are unclear, one possibility is due to *Borrelia burgdorferi* persisters that are not effectively killed by current antibiotics such as doxycycline or amoxicillin used to treat Lyme disease. A previous study showed that four rounds of ceftriaxone pulse dosing treatment eradicated *B. burgdorferi* persisters *in vitro* using a relatively young late log phase culture (5 day old). In this study, we investigated if ceftriaxone pulse dosing could also eradicate *B. burgdorferi* persisters in older stationary phase cultures (10 day old) enriched with more resistant microcolony form of persisters. We found that ceftriaxone pulse dosing could only eradicate planktonic log phase *B. burgdorferi* spirochetal forms and round body forms but not more resistant aggregated biofilm-like microcolony persisters enriched in stationary phase cultures. Moreover, we found that not all drugs are suitable for pulse dosing, with bactericidal drugs ceftriaxone and cefuroxime being more appropriate for pulse dosing than bacteriostatic drug doxycycline and persister drug daptomycin. We also showed that drug combination pulse dosing treatment was more effective than single drug pulse dosing. Importantly, we demonstrate that pulse dosing treatment impaired the activity of the persister drug daptomycin and its drug combination against *B. burgdorferi* persisters and that the most effective way to kill the more resistant biofilm-like microcolonies is the daptomycin/doxycycline/ceftriaxone triple drug combination without pulse dosing. Our findings indicate pulse dosing may not always work as a general principle but rather depends on the specific drugs used, with cidal drugs being more appropriate for pulse dosing than static or persister drugs, and that drug combination approach with persister drugs is more effective at killing the more resistant microcolony form of persisters than pulse dosing. These observations may have implications for more effective treatment of Lyme disease. Future studies are required to validate these findings in animal models of *B. burgdorferi* persistence.

## Introduction

Lyme disease, caused by *Borrelia burgdorferi*, is the most common vector-borne disease in the United States with an estimated 300,000 cases in 2013 (CDC, 2015a). The infection is transmitted to humans by tick vectors that feed upon rodents, reptiles, birds, and deer (Radolf et al., 2012). In the early stage of Lyme disease, patients often have localized erythema migrans skin lesions, but late stage Lyme disease is a disseminated multi-system disorder with signs and symptoms including arthritis, carditis, and neurologic impairment (CDC, 2015a). The majority of Lyme disease patients can resolve their symptoms if treated promptly with doxycycline, amoxicillin, or cefuroxime (Wormser et al., 2006). However, at least 10–20% of Lyme disease patients have lingering symptoms such as fatigue, muscular and joint pain, and neurologic impairment, 6 months after the standard 2–4 week antibiotic treatment, a collection of symptoms called Post-Treatment Lyme Disease Syndrome (PTLDS; CDC, 2015b).

While the cause of PTLDS is unknown, there are several theories, including co-infections (Swanson et al., 2006), autoimmune response (Steere et al., 2001), immune response to continued presence of antigenic debris (Bockenstedt et al., 2012), as well as *B. burgdorferi* persisters that are not killed by the current antibiotics (Hodzic et al., 2008, 2014; Embers et al., 2012). Using xenodiagnosis and quantitative PCR, various studies have found evidence of *B. burgdorferi* persistence in dogs (Straubinger et al., 1997), mice (Hodzic et al., 2008, 2014), monkeys (Embers et al., 2012), and humans (Marques et al., 2014) after antibiotic treatment, though no viable bacteria could be cultured.

Recently, *B. burgdorferi* has been shown to develop persisters in stationary phase cultures *in vitro*, which are tolerant to the antibiotics used to treat Lyme disease (Feng et al., 2014a, 2015a; Caskey and Embers, 2015; Sharma et al., 2015). These persister bacteria have an altered gene expression profile, which may underlie their phenotypic drug tolerance (Feng et al., 2015c). In log phase cultures (3–5 day old), *B. burgdorferi* is primarily in motile spirochetal form and is highly susceptible to current Lyme antibiotics doxycycline and amoxicillin (Feng et al., 2014a, 2015a). However, in stationary phase cultures (7–15 day old), increased numbers of atypical morphological variants such as round bodies and aggregated biofilm-like microcolonies develop (Feng et al., 2014a, 2015a). These atypical forms can be considered part of the heterogeneous persisters (Zhang, 2014) as they have high tolerance to doxycycline and amoxicillin compared to growing spirochetal forms (Feng et al., 2014a, 2015a). Therefore, stationary phase cultures (7–15 day old) which are enriched in persisters have been used as a surrogate persister model for high-throughput drug screens against persister populations (Feng et al., 2014a, 2015a,b,d) and have been shown to have overlapping persister populations with amoxicillin tolerant round body persisters (Feng et al., 2016a).

Drugs with high activity against the *B. burgdorferi* stationary phase persisters were identified through screens of FDA approved drug library and NCI compound library (Feng et al., 2014a, 2015a,b,d). Among them, daptomycin, a lipopeptide antibiotic targeting bacterial cell membranes used to treat MRSA, has the highest activity against *B. burgdorferi* persisters (Feng et al., 2014a). Although the anti-persister drugs such as daptomycin are more active than the current Lyme antibiotics such as doxycycline or amoxicillin against *B. burgdorferi* persisters (Feng et al., 2014a), they alone could not completely eradicate the more resistant biofilm-like microcolonies and a drug combination approach is required to do so (Feng et al., 2015a). The more effective drug combination approach to eradicate biofilm-like microcolonies is consistent with the drug combination principle for treatment of persistent infections like tuberculosis (Zhang et al., 2012; Zhang, 2014). In a recent study, ceftriaxone pulse dosing was shown to completely eradicate *B. burgdorferi* persisters using a relatively young 5 day old late log phase culture (Sharma et al., 2015), which is known to primarily consist of spirochetal form that is more susceptible to antibiotics. It is unclear whether the pulse dosing approach is able to eradicate the more resistant microcolony form of persisters (Feng et al., 2015a). In this study, we evaluated the pulse dosing approach with ceftriaxone and also other Lyme antibiotics and drug combinations using an older stationary phase culture (10 day old) enriched with more resistant biofilm-like microcolonies as well as a 5 day old late log phase culture as a control to determine their ability to eradicate *B. burgdorferi* persisters. We found that four rounds of pulse dosing treatment with ceftriaxone could eliminate mainly the planktonic spirochetal form of *B. burgdorferi* in the log phase and the stationary phase culture, but failed to eradicate the aggregated microcolony form of *B. burgdorferi* persisters in the stationary phase culture.

## Materials and Methods

### Strain, Media, and Culture

Low passaged *B. burgdorferi* strain B31 5A19 was kindly provided by Monica Embers (Caskey and Embers, 2015). The *B. burgdorferi* B31 strain was cultured in BSK-H medium (HiMedia Laboratories Pvt. Ltd.), with 6% rabbit serum (Sigma–Aldrich) in microaerophilic incubator (33°C, 5% CO_2_) without antibiotics. After incubation for 7–10 days, 1 ml stationary phase *B. burgdorferi* culture was transferred into 1.5 ml Eppendorf tubes for evaluating the effect of antibiotic treatment.

### Antibiotics

Doxycycline (Dox), cefuroxime, ceftriaxone (CefT; Sigma–Aldrich, USA), and daptomycin (Dap) (AK Scientific Inc., USA) were dissolved in suitable solvents as suggested by the Clinical and Laboratory Standards Institute (CLSI) to obtain stock solutions. The antibiotic stocks were filter-sterilized by 0.2 μm filter. Then the stocks were stored at -20°C.

### Microscopy Techniques

The *B. burgdorferi* cultures were examined using a Zeiss AxioImager M2 microscope with epifluorescence illumination. Pictures were taken using a SPOT slider camera. The SYBR Green I/PI viability assay was performed to assess cell viability using the ratio of green/red fluorescence to determine the live:dead cell ratio, respectively, as described previously (Feng et al., 2014b, 2015a). This residual cell viability reading was confirmed by analyzing three representative images of the bacterial culture using epifluorescence microscopy. Image Pro-Plus software was used to quantitatively determine the fluorescence intensity (Shopov et al., 2000).

### Pulse Dosing Treatment

Aliquots of a 10 day old stationary phase culture (1 ml) of *B. burgdorferi* and a 5 day old late log phase culture (as a control) were treated with single antibiotic or drug combinations (all at 5 μg/ml) for 5 days in Eppendorf tubes, which is considered the first round of antibiotic treatment. Then the cultures were washed twice and resuspended in fresh BSK-H medium for recovery for 1 day at 33°C without shaking. Then the culture was treated again with drugs or drug combinations for another 5 days to give the second round of treatment. This was repeated for a total of four rounds of treatment. To determine the effect of antibiotics, 100 μl of treated *B. burgdorferi* culture was transferred to a 96-well plate and the SYBR Green I/PI viability assay was used to assess the live and dead cells after antibiotic exposure as described (Feng et al., 2014a). Briefly, 10 μl of SYBR Green I/PI staining mixture was added to each well and mixed thoroughly. The plates were incubated at room temperature in the dark for 15 min followed by plate reading at excitation wavelength at 485 nm and fluorescence intensity at 535 nm (green emission), and 635 nm (red emission) in microplate reader (HTS 7000 plus Bio Assay Reader, PerkinElmer Inc., USA). With least-square fitting analysis, the regression equation and regression curve of the relationship between percentage of live bacteria and green/red fluorescence ratios were obtained. The regression equation was used to calculate the percentage of live cells in each well of the 96-well plate. At the same time, microscopy followed by SYBR Green I/PI staining was used to confirm the results of plate reader as previously described (Feng et al., 2014a).

### Subculture Studies to Assess Viability of Antibiotic-Treated *B. Burgdorferi*

After four rounds of pulse dosing treatment, the bacteria were spun down and washed twice with 1 mL fresh BSK-H medium. The cell pellet was resuspended in 600 μl BSK-H medium, and a 200 μl aliquot was used to inoculate a new tube of 1 ml fresh BSK-H medium for subculture. The cultures were allowed to grow for up to 21 days, when they were evaluated for regrowth with viable cells using the SYBR Green I/PI assay and epifluorescence microscopy as previously described (Feng et al., 2015a).

## Results

### Ceftriaxone Pulse Dosing Sterilized Log Phase Culture But Fails to Do so for Stationary Phase *B. burgdorferi* Culture Enriched with More Resistant Biofilm-Like Microcolonies

**FIGURE 1:**
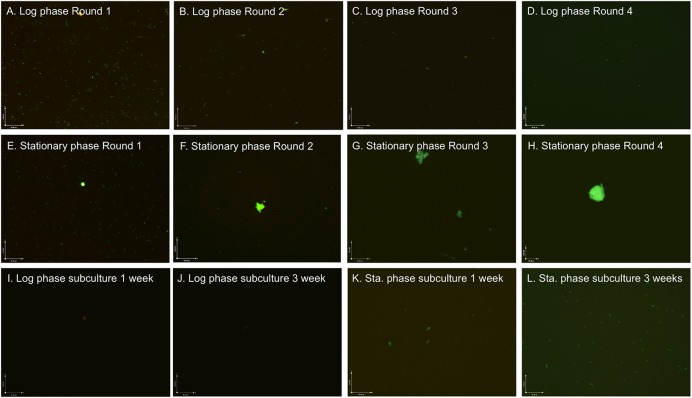
**Effect of ceftriaxone pulse dosing on 5 day old log phase and 10 day old stationary phase *Borrelia burgdorferi* cultures.** Log phase **(A–D, I,** and **J)** and stationary phase **(E–H, K,** and **L)** *B. burgdorferi* cultures were treated with pulse dosing of 5 μg/mL ceftriaxone as described in the section “Materials and Methods” followed by staining by SYBR Green I/PI assay and fluorescence microscopy at 100× magnification. Abbreviation: Sta., stationary.

Ceftriaxone is one of the best clinically used antibiotics for treating late stage Lyme disease (Dattwyler et al., 1988) and has been shown to have good activity against *B. burgdorferi* persisters (Feng et al., 2014a). A previous study showed that four rounds of ceftriaxone pulse dosing treatment could eradicate late log phase culture (5 day old) below the limit of detection (Sharma et al., 2015). To determine if the pulse dosing treatment is able to eradicate older stationary phase culture containing more resistant microcolony form of persisters, we compared pulse dosing treatment with ceftriaxone (5 μg/ml) on a 10 day old stationary phase culture of *B. burgdorferi* and on a 5 day old late log phase culture as a control using procedures as described previously (Sharma et al., 2015). As shown in our previous studies (Feng et al., 2014a, 2015a), the spirochetal form dominated the 5 day old log phase culture, with few round body forms and rare microcolony forms, whereas truly stationary phase cultures developed increasing proportions of variant forms such as round bodies and microcolonies that are more resistant to antibiotics. As the round of pulse dosing treatment with ceftriaxone increased, the number of spirochetal forms and round body forms decreased in the log phase culture (**Figures 1A–D**). We noted after the first round of ceftriaxone treatment the number of planktonic spirochetes decreased dramatically while the number of round body forms increased slightly presumably due to conversion of spirochetal form to round body forms (Feng et al., 2016a) in both the 5 day old late log phase and the 10 day old stationary phase cultures. Two and three rounds of treatment could kill both spirochetal form and round body forms, and the fourth round of treatment eliminated nearly all planktonic *B. burgdorferi* cells but still could not eliminate the aggregated microcolony form in the 10 day stationary phase culture (**Figure 1H**). The large portions of microcolony forms (Feng et al., 2015a) not killed by pulse dosing treatment (**Figures 1E–H**), could be seen by SYBR Green I/PI assay with green fluorescence despite four rounds of ceftriaxone pulse dosing (**Figure 1H**). In the meantime, we used drug free control in the four rounds of pulse dosing treatment to eliminate the possible influence of frequent centrifugation and washes on loss of bacteria and found that these steps did not significantly affect the results of the pulse dosing experiment.

To confirm the results of the SYBR Green I/PI microscopy assay, we performed subculture test in fresh BSK-H medium as in our previous study (Feng et al., 2015a). The results showed that the 5 day log phase culture treated with four rounds of ceftriaxone pulse dosing did not regrow after 7 and 21 days subculture (**Figures 1I,J**). The four rounds of ceftriaxone pulse dosing treated 10 day old stationary phase culture did not show signs of regrowth after 7 day subculture despite the presence of some microcolony forms of *B. burgdorferi* (**Figure 1K**). However, after 21 day subculture, about 3 × 10^6^ regenerative spirochetes per milliliter in the stationary phase subculture were observed (**Figure 1L**). These results suggest that although four rounds of ceftriaxone pulse dosing treatment could eliminate planktonic log phase *B. burgdorferi* spirochetal forms and even round body forms, it failed to eradicate the more resistant aggregated microcolony forms of *B. burgdorferi* in the stationary phase culture.

### Drugs Differ Considerably in Their Ability to Sterilize *B. Burgdorferi* Cultures in Pulse Dosing Treatment

**FIGURE 2:**
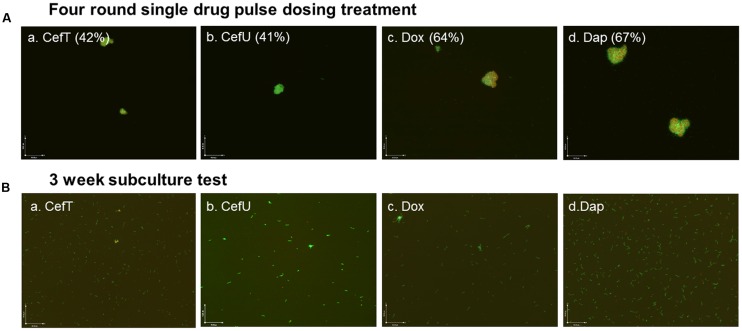
**Representative images of a 10 day old *B. burgdorferi* stationary phase culture treated with four rounds of pulse dosing treatment with different single drugs (Aa–d)** and subcultured for 21 days in fresh BSK-H medium **(Ba–d)**. Stationary phase *B. burgdorferi* culture (10 day old) was treated with different single drug (5 μg/ml) pulse dosing treatment as described in the section “Materials and Methods,” followed by staining by SYBR Green I/PI assay and fluorescence microscopy at 100× magnification. The percentage of residual viable cells is shown in brackets. The percentage of residual viable cells was calculated according to the regression equation and green/red fluorescence ratios. None of the single drugs were able to eradicate the microcolony forms as shown by either SYBR Green I/PI staining or subculture studies. Abbreviations: CefT, ceftriaxone; CefU, cefuroxime; Dox, doxycycline; Dap, daptomycin.

Since ceftriaxone pulse dosing could eradicate *B. burgdorferi* in log phase cultures but not in stationary phase cultures, we wanted to investigate how persister active drug daptomycin and other Lyme antibiotics such as doxycycline and cefuroxime behave in the pulse dosing experiment. We subjected a 10 day old stationary phase culture of *B. burgdorferi* to daptomycin, doxycycline, and cefuroxime all at 5 μg/ml with ceftriaxone as a positive control to four rounds of pulse dosing treatment as described in the section “Materials and Methods.” Our results showed that daptomycin was less effective (residual viability 67%) than ceftriaxone (residual viability 42%) and had comparable activity with doxycycline (residual viability 64%) after four rounds of pulse dosing treatment (**Figure 2A**). Interestingly, cefuroxime had comparable activity (41% residual viable cells) as ceftriaxone (42% residual viable cells) after four rounds of pulse dosing treatment (**Figure 2A**). In subculture studies, all the cultures subjected to four rounds of single drug pulse dosing treatment with daptomycin, or doxycycline, or cefuroxime, or ceftriaxone grew back after 21 days (**Figure 2B**), indicating single antibiotic pulse dosing is not able to eradicate stationary phase *B. burgdorferi* cultures.

### Drug Combination Is More Effective than Single Drug in Pulse Dosing Treatment

**FIGURE 3:**
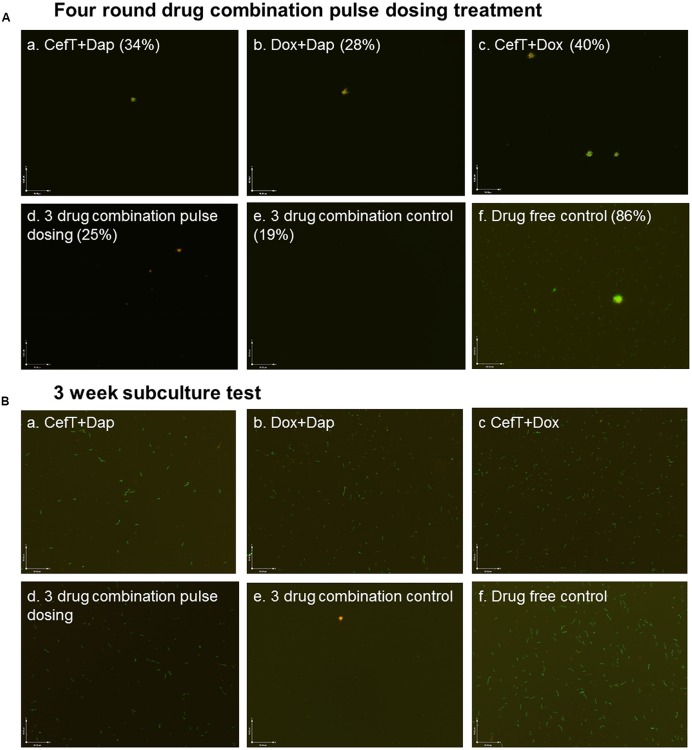
**Representative images of a 10 day old *B. burgdorferi* stationary phase culture treated with drug combination pulse dosing treatment (Aa–f)** and subcultured for 21 days in fresh BSK-H medium **(Ba–f)**. Stationary phase *B. burgdorferi* culture (10 day old) was treated with pulse dosing treatment of two drug combinations (CefT+Dap; Dox+Dap, CefT+Dox) or the three drug combination (ceftriaxone/doxycycline/daptomycin; 5 μg/ml) followed by staining with SYBR Green I/PI assay and fluorescence microscopy at 100× magnification. The percentage of residual viable cells is shown in brackets. The percentage of residual viable cells was calculated according to the regression equation and green/red fluorescence ratios as described in the section “Materials and Methods.” Abbreviations: CefT, ceftriaxone; Dox, doxycycline; Dap, daptomycin.

Despite the good activity of ceftriaxone pulse dosing treatment against log phase culture of *B. burgdorferi* (**Figures 1A–D**) it had poor activity against the most resistant microcolony form of persisters in stationary phase cultures (**Figures 1E–H**). Our previous studies suggest that drug combination treatment is more effective than single drug treatment and could eradicate the *B. burgdorferi* microcolony forms *in vitro* (Feng et al., 2015a, 2016a,b) and that daptomycin showed best activity against stationary phase and round body form of *B. burgdorferi* (Feng et al., 2014a, 2016a). The daptomycin/doxycycline combined with cefoperazone or cefuroxime is the most effective drug combination which could eradicate even the most resistant aggregated microcolony form of *B. burgdorferi* enriched in stationary phase cultures (Feng et al., 2015a, 2016b). To determine if daptomycin drug combinations could eradicate the stationary phase *B. burgdorferi* in the pulse dosing treatment, we tested drug combination pulse dosing treatment on a 10 day old *B. burgdorferi* stationary phase culture enriched with microcolony forms at 5 μg/ml of each drug (close to the plasma concentration). We found that the daptomycin combination pulse dosing treatment (residual viability 25–34%) were obviously better than the single drug pulse dosing treatment (**Figures 3A** and **2A**). On the other hand, the ceftriaxone and doxycycline combination did not produce significantly better activity (residual viability 40%; **Figure 3Ac**) than the ceftriaxone alone (residual viability 42%; **Figure 2Aa**) after four rounds of pulse dosing treatment.

In three drug combination (ceftriaxone/doxycycline/dapto-mycin) pulse dosing, the treatment showed better activity (residual viability 25%) than two drug combination pulse dosing treatments (residual viability 40–28%; **Figure 3A**). Among the two drug combination pulse dosing treatments, doxycycline/daptomycin combination showed the best activity (residual viability 28%), and ceftriaxone/daptomycin combination showed better activity (residual viability 34%), while the ceftriaxone/doxycycline showed least activity (residual viability 40%). However, we found that the above drug combination pulse dosing treatments all failed to eradicate the microcolony forms (**Figure 3A**). Interestingly, we found the triple drug combination treatment (ceftriaxone/doxycycline/daptomycin) without pulse dosing was the most effective among all drug combinations tested and was more active (residual viability 19%) than the same triple drug combination with pulse dosing treatment (residual viability 42%). The three drug combination treatment without pulse dosing eradicated virtually all visible *B. burgdorferi* cells including the aggregated microcolony form (**Figure 3A**), while after four rounds of the three drug combination pulse dosing treatment some aggregated microcolony forms were still observe persisting (**Figure 3A**).

To confirm the results of the SYBR Green I/PI assay, we performed subculture experiment on the drug combination pulse dosing treated 10 day old *B. burgdorferi* stationary phase cultures. The results showed that cultures treated with two drug combination pulse dosing (CefT+Dap; Dox+Dap, CefT+Dox) all grew up after 21 day subculture (**Figures 3Ba–c**). Interestingly, the triple drug combination (ceftriaxone/doxycycline/daptomycin) pulse dosing failed to eradicate *B. burgdorferi* in the subculture test as shown by regrowth of visible spirochetal organisms after 21 days (**Figure 3Bd**), by contrast, the same triple drug combination (ceftriaxone/doxycycline/daptomycin) without pulse dosing completely eradicated all *B. burgdorferi* including the most resistant microcolonies as demonstrated by lack of any regrowth in subculture (**Figure 3Be**).

## Discussion

A previous study showed that ceftriaxone could eliminate all cells including persisters in a 5 day old *B. burgdorferi* culture after four rounds of pulse dosing treatment (Sharma et al., 2015). In this study, we were able to confirm this finding using the same protocol with a relatively young 5 day old late log phase *B. burgdorferi* culture. However, we found that although four rounds of ceftriaxone pulse dosing treatment could eliminate nearly all planktonic spirochetes in the 5 day old culture and even those in a 10 day old stationary phase *B. burgdorferi* culture, it failed to eradicate the more resistant aggregated biofilm-like microcolonies enriched in stationary phase cultures (**Figure 1**). Our previous studies demonstrated that a 5 day old culture is quite young and could be considered late log phase (Feng et al., 2014a) rather than “stationary phase” as used in a previous study (Sharma et al., 2015) and is mainly consisting of spirochetal organisms that are more susceptible to antibiotics. In contrast, 7–10 day old cultures of *B. burgdorferi* can be considered true stationary phase and contain higher proportions of aggregated microcolony forms, which are much more tolerant to antibiotics than the spirochetal forms (Feng et al., 2015a). Thus, it is not surprising that ceftriaxone failed to eradicate the more resistant biofilm-like microcolonies of *B. burgdorferi* as shown in this study (**Figure 1**), as heterogeneous microcolonies have previously been shown to be highly tolerant to antibiotics including single drugs as well as many drug combinations except daptomycin/doxycycline/cefoperazone (Feng et al., 2015a). In addition, a short 1 day recovery in fresh medium used in this study per the previous pulse dosing protocol (Sharma et al., 2015) may not be sufficiently long to allow all the dormant forms in aggregated microcolonies to wake up and become susceptible to ceftriaxone. Indeed, the time interval in between pulse dosing treatment needed to allow the persisters to revive and become susceptible to antibiotics is hard to determine not only for *in vitro* persisters but also for *in vivo* persisters in patients, considering the heterogeneity of the bacteria and the disease and individual differences in immune control, which could pose significant challenges to the use of pulse dosing in clinical settings (Zhang, 2014).

An important observation of this study is that pulse dosing may not always work as a principle against persisters but rather its effect depends on the particular drugs being used and that not all drugs are suitable for pulse dosing. We found that daptomycin, which is the most effective antibiotic against *B. burgdorferi* persisters (Feng et al., 2014a, 2016a), fared poorly in pulse dosing experiments either singly (**Figure 2A**) or in drug combinations (**Figure 3A**), as it had even less activity than the ceftriaxone and doxycycline in the pulse dosing treatment (**Figure 2A**). Despite its excellent activity against non-growing *B. burgdorferi* persisters (Feng et al., 2014a), daptomycin is known to have limited activity against growing spirochetal forms as shown by a relatively high MIC (12.5–25 μg/ml) compared to ceftriaxone and doxycycline (Feng et al., 2014a). When used at a concentration under its MIC (5 μg/ml) as in this study, daptomycin is expected to have little activity against growing *B. burgdorferi* thus explaining its poor activity in the pulse dosing treatment. However, we do find better activity when daptomycin was combined with antibiotics such as ceftriaxone and doxycycline which act on actively growing bacteria even in the pulse dosing experiment (**Figure 2A**). The basic idea of pulse dosing treatment is to allow non-growing antibiotic tolerant persisters formed after drug treatment to recover and become growing spirochetes so they become susceptible to drugs again. Thus, pulse dosing seems to work well with cidal antibiotics as shown with ceftriaxone (Sharma et al., 2015) (**Figure 1**) and cefuroxime (**Figure 2A**) but not with static drugs like doxycycline (Caskey and Embers, 2015) (**Figure 2Ac**) and persister drugs such as daptomycin as shown in this study (**Figure 2A**). Our findings suggest that only bactericidal antibiotics like cephalosporins may be suitable for pulse dosing but not bacteriostatic agents or persister drugs. These observations may have important implications on clinical use of pulse dosing for treatment of persistent infections like Lyme disease.

Given the role of pulse dosing with cidal antibiotics, its effect as a single drug on the more resistant microcolonies is limited and could not eradicate aggregated biofilm-like structures (**Figure 1**). In our previous studies, we found that drug combinations containing persister drug daptomycin are more effective against *B. burgdorferi* persisters than single drug treatment (Feng et al., 2015a, 2016b). Consistent with this, we found that the drug combination pulse dosing treatment also showed more activity against stationary phase *B. burgdorferi* than single drug pulse dosing (**Figures 3A and 2A**). This is supported by microscopic observations that the amount and the size of the aggregated microcolonies were significantly reduced after the drug combination pulse dosing treatment compared with the single drug pulse dosing treatments (**Figure 3A**). In addition, three drug combination pulse dosing treatment was more effective than two drug combination pulse dosing treatment (**Figure 3A**). However, either the two drug combination or even three drug combination pulse dosing were still unable to eradicate the more resistant microcolony forms, and the residual microcolonies grew up again in the subculture test without antibiotics (**Figure 3B**).

We previously showed that daptomycin/doxycycline combined with cefoperazone or cefuroxime as a triple drug combination has the best activity against stationary phase *B. burgdorferi* cultures and could completely eradicate all bacteria including the most resistant aggregated microcolony form of persisters (Feng et al., 2015a, 2016b). In this study, we found the ceftriaxone or cefuroxime could also replace cefoperazone in the daptomycin/doxycycline combination to kill all the stationary phase *B. burgdorferi* cells (**Figure 3**). Unexpectedly, however, the triple drug combination daptomycin/doxycycline/ceftriaxone pulse dosing treatment turned out to be less effective than the continuous triple drug combination treatment as shown by SYBR Green I/PI assay as well as the subculture test (**Figure 3**). Thus pulse dosing treatment compromised the activity of daptomycin-containing drug combinations through reducing the activity of daptomycin as shown in **Figure 2Ad**. Further studies are required to validate the findings of these *in vitro* studies *in vivo* and compare the utility of the pulse dosing and the drug combination treatment in eradication of borrelia persistence phenomenon in animal models.

## Conclusion

We found that four rounds of ceftriaxone pulse dosing could eradicate planktonic log phase *B. burgdorferi* spirochetal forms and round body forms but not the more resistant aggregated biofilm-like microcolony forms in the stationary phase culture. In addition, we showed that not all drugs are appropriate for pulse dosing, with ceftriaxone and cefuroxime being more suitable for pulse dosing than bacteriostatic drug doxycycline and persister drug daptomycin. This study also demonstrated that drug combination pulse dosing treatment is more effective than single drug pulse dosing. Importantly, we found that pulse dosing could impair the activity of the persister drug daptomycin and its drug combination on *B. burgdorferi* persisters and that the more effective way to kill the aggregated biofilm-like microcolony form of persisters is the triple daptomycin drug combination without pulse dosing. Future studies are needed to validate these findings in animal studies and clinical trials.

## Author Contributions

YZ conceived the experiments; JF, SZ, and WS performed the experiments; JF and YZ analyzed the data; JF and YZ wrote the paper.

## Conflict of Interest Statement

The authors declare that the research was conducted in the absence of any commercial or financial relationships that could be construed as a potential conflict of interest. The reviewer NBDC and handling Editor declared their shared affiliation and the handling Editor states that the process nevertheless met the standards of a fair and objective review.
